# Are there clinical and subclinical/pathological forms of Paget’s disease of the breast?

**DOI:** 10.3389/fonc.2023.1287882

**Published:** 2023-11-28

**Authors:** Rafael José Fábio Pelorca, Idam de Oliveira-Junior, René Aloisio da Costa Vieira

**Affiliations:** ^1^ Postgraduate Program in Tocogynecology, Botucatu School of Medicine, Botucatu, SP, Brazil; ^2^ Postgraduate Program in Oncology, Barretos Cancer Hospital, Barretos, SP, Brazil; ^3^ Department of Mastology and Breast Reconstruction, Barretos Cancer Hospital, Barretos, SP, Brazil; ^4^ Department of Surgical Oncology, Division of Breast Surgical Oncology, Muriaé Cancer Hospital, Muriaé, MG, Brazil

**Keywords:** breast neoplasms, breast-conserving surgery, oncoplastic surgery, Paget’s disease of the breast, neoplasm staging

## Abstract

**Introduction:**

Breast disease management has changed over recent decades, related to molecular subtype, oncoplastic surgery and targeted therapies. Nevertheless, literature on Paget’s disease of the breast (PDB), initially described as a clinical entity and now considered a multifocal/multicentric disease.

**Methods:**

PDB was classified as clinical in the presence of areolar abnormalities and as subclinical/pathological in all other cases. Clinical and prognostic data were evaluated and compared between the different presentation forms. Statistics comprised descriptive analysis, inter-group comparison (chi-square and Mann-Whitney tests) and overall and cancer-specific survival rates (Kaplan-Meier method and the log-rank test).

**Results:**

Of 85 patients included in this series, PDB was clinical in 58.8%. Overall, 27.1% had stage 0 and 92.9% had multifocal/multicentric disease. Most patients (83.5%) had the HER2 or luminal HER2 molecular subtype. Patients with clinical PDB had a higher rate of *in situ* disease (p=0.028) and were more likely to undergo breast-conserving surgery (p<0.001). Most of the 43 patients with HER2 invasive disease received anti-HER therapy. Mean follow-up time was 71.2 ± 43.3 months. Cancer-specific actuarial survival at 60 and 120 months was 92.3% and 83.1%, respectively. Survival was unaffected by the clinical form of PDB (p=0.275), anti-HER therapy (p=0.509) or oncoplastic surgery (p=0.821). Conversely, clinical stage affected survival significantly (p ≤ 0.001).

**Conclusion:**

PDB is a rare condition associated with multifocality/multicentricity and HER2 overexpression. Cases of clinical disease and those of subclinical/pathological disease differ significantly. Further studies are required to evaluate the clinical/areolar disease and the impact of advances in breast disease management on PDB.

## Introduction

The first report on Paget’s disease of the breast (PDB) described it from a clinical viewpoint as a condition associated with abnormalities of the nipple (1). However, population-based studies (2–5) using pathology databases have provided further understanding of its incidence, showing that the disease may be involved in 1 to 4.3% of cases of breast cancer (5). Due to its relative rarity, single-institution series are limited to approximately 50 patients (6–9). Notwithstanding, the number of case series or, more commonly, of case reports has increased in the literature. Few studies involving larger series of patients have been conducted in Brazil (10–12).

PDB is a form of nipple pathology, although in its early stages the nipple-areolar complex (NAC) may appear normal, constituting a subclinical form of the disease (13). The condition is associated with other tumors in which a high rate of multifocality, affecting up to 90% of cases, is found (9, 13).

Recently, molecular subtype evaluation showed a strong association between PDB and HER-2 positivity (3, 12, 14), however, the effect of anti-HER2 therapy on this disease has yet to be clarified. Initially considered a tumor with a good prognosis, some reports in the literature now suggest a potentially negative prognosis (2, 3), a hypothesis that has yet to be fully investigated.

Over recent decades, radical changes have occurred in the diagnosis, analysis and treatment of breast cancer, including surgical treatment, chemotherapy and immunotherapy. Mammography has become digital; magnetic resonance imaging (MRI) is now part of the diagnostic arsenal; molecular subtypes form part of routine clinical practice even in cases of *in situ* disease; the tumor-node-metastasis (TNM) staging system is used to estimate prognosis; oncoplastic surgery has optimized locoregional treatment; and anti-HER2 therapy has been integrated into daily clinical practice. Consequently, PDB needs to be reevaluated in the light of these new developments, and the protocol of the present study was designed for this purpose.

## Materials and methods

The institute’s internal review board approved the protocol of this retrospective study under reference number 657293, CAAE 31046314.5. The study included patients receiving care at a tertiary cancer hospital between 2000 and 2021. All cases had a confirmed pathological diagnosis of PDB as registered in the institute’s pathology database. A standardized form was used to retrospectively collect clinical, pathological and imaging data from the patient charts, as well as data related to treatment, recurrence and death.

To analyze histological type, four groups were formed according to tumor characteristics: PDB affecting only the NAC, PDB associated with ductal carcinoma *in situ* (DCIS), PDB associated with invasive disease, and PDB associated with DCIS and invasive carcinoma. Evaluation of tumor size took into consideration the greatest diameter irrespective of whether the disease was *in situ* or invasive.

Clinical PDB was defined as cases that presented with eczema, erosion or ulceration of the nipple, with all other cases being considered pathological or subclinical PDB. These two groups were separated for the purpose of analysis (**Table 1** and **Supplementary Table 1**).

**Table 1:** Clinicopathological variables.

Variable	Category	Clinical	PDB		p
		Present	Absent	Total	
Time of history	months	39,7 ± 34,5	51,0 ± 34,5	44,55±34,7	0,074
Age	years	53,1±12,3	49,0±13,4	52,2±13,3	0,266
Tumor full size	cm	3,5±2,9	4,9±3,6	4,1±3,3	0,068
Tumor invasive size	cm	1,4±3,2	2,0±3,3	2,5±2,8	0,129
Follow-up time	months	72,2±45,1	65,5±40,6	71,2±43,3	0,330
Age	<40 years	5	7	12 (14,1%)	0,398
	40 - 50 years	17	11	28 (32,9%)	
	50 - 60 years	11	9	20 (23,5%)	
	60 - 69 years	13	4	17 (20%)	
	≥70 years	4	4	8 (9,4%)	
Treatment	2006 - 2009	7	4	11 (12,9%)	0,072
period	2010 - 2013	20	8	28 (32,9%)	
	2014 - 2017	5	11	16 (18,8%)	
	2018 - 2021	18	12	30 (35,3%)	
Clinical	0	19	4	23 (27,1%)	0,028
Stage TNM	I	12	8	20 (23,5%)	
	II	6	11	17 (20%)	
	III	13	11	24 (28,2%)	
	IV	0	1	1 (1,2%)	
Prognostic	0	19	4	23 (27,1%)	0,054
Stage TNM	I	13	11	20 (23,5%)	
	II	7	10	17 (20,0%)	
	III	11	9	24 (28,2%)	
	IV	0	1	1 (1,2%)	
Molecular	DCIS	18	3	21 (25,3%)	0.033
subtype	Lum. Her-	4	8	12 (14,5%)	
	Lum. B Her+	9	8	17 (20,5%)	
	Her+	15	11	26 (31,3%)	
	Triple negative	3	4	7 (8,4%)	
Histological	PDB	6	0	6 (7,1%)	0,045
subtype	PDB+ *DCIS*	12	4	16 (18,8%)	
	PDB + IC	26	23	49 (57,6%)	
	PDB + DCIS + IC	6	8	14 (16,5%)	

PDB, Paget Disease of the Breast; TN, triple negative; Lum., Luminal; DCIS, ductal carcinoma in situ; ID, invasive carcinoma.

The time of follow-up was defined as the period between the first consultation and the last information obtained at the institute, with data being updated using the hospital cancer registry. Cases in which the patient failed to return for a scheduled visit within a period exceeding twice the expected time were considered lost to follow-up.

Local recurrence was defined as disease recurrence in the homolateral breast or chest wall. Overall survival and cancer-specific survival were estimated. For the patients who died, the cause of death was determined, with death resulting from breast cancer being considered disease-related death. If death occurred from another cause, this was taken into consideration when estimating overall survival; however, that patient was considered to be alive for the purpose of evaluating cancer-specific survival.

Descriptive statistics were performed for categorical and continuous variables. The continuous variables were presented as medians and standard deviations ( ± SD), with the difference or lack of difference between the clinical and pathological/subclinical forms being determined. The chi-square test was used to compare variables between the groups, with Fisher’s exact test being used when a class included fewer than 5 variables. The Mann-Whitney test was used to compare continuous variables. The Kaplan-Meier method was used to estimate survival over time, while the log-rank test was used to compare survival between the groups. P-values <0.05 were considered statistically significant. IBM SPSS, version 20 for Mac^®^, was used to store the data and perform the statistical analysis.

## Results

During the established study period between 2000 and 2021, 85 women with PDB were identified, with 87.1% of these women having been diagnosed and treated after 2010. Mean age was 52.2 ± 13.3 years, and 56.5% of these women were in the 40-59-year age bracket.

At clinical presentation (**Supplementary Figure 1**), the signs/symptoms most associated with the disease were localized swelling (57.0%), eczema (40.7%) and ulceration of the nipple (27.9%).

Regarding histopathology, the disease was confined to the NAC in only 7.1% of cases, while PDB was associated with DCIS in 18.8%, with invasive carcinoma in 57.6% and with DCIS and invasive carcinoma in 16.5%. From a clinical point of view, the areolar disease was perceptible in 58.8% of cases and a palpable tumor was present in 56.5%. Although bilateral disease was present in 4.7% of cases, PDB was unilateral in all the patients in this study, and was predominantly situated on the right side (58.8%). Mean tumor size was 4.1 ± 3.3 cm.

Mammography was performed in 85.9% of patients, with some form of abnormality being detected in 87.7% of these cases. The most commonly found abnormality was micro-calcifications (58.9%), followed by nodulation (37%). Ultrasonography of the breast was performed in 79 patients (92.9%), with the most common abnormality found being a solid lump in 72.2% of patients. Twenty-three patients (27.1%) underwent MRI of the breast, with tumors being detected in 69.6% of cases and thickening of the NAC in 43.5% of cases. There was no statistically significant difference between the different groups of PDB (clinical or subclinical/pathological) in relation to the frequency of imaging tests: mammography (p=0.540), ultrasonography of the breast (p=0.393) and MRI of the breast (p=0.321). Patients considered to have clinical PDB were more likely to have a finding of skin thickening (p=0.04) at mammography and of abnormalities of the NAC at ultrasonography (p=0.07).

Regarding clinical staging, 27.1% of cases were considered as stage 0, 23.5% stage I, 20% stage II and 28.2% stage III, with one patient being found to have metastatic disease at diagnosis (1.2%). Differences were observed between clinical and prognostic TNM (p<0,001 (**Supplementary Table 2**). At pathology, the mean size of the *in situ* tumors was 7.2 cm and the mean size of the invasive tumors was 3.1 cm. Cases of PDB classified as clinical were associated with earlier stages (p=0.028). **Table 1** and **Supplementary Table 1** report clinical, pathological, and radiological variables.

For the analysis of molecular subtype (**Supplementary Table 3**), patients were separated into those with *in situ* disease (25.3%) and those with invasive disease. In two cases, immunohistochemistry was not performed. In the 67 patients with invasive disease, 64.2% had HER2 expression, with 38.8% (26/67) having HER2 over-expression and 25.4% (17/67) luminal HER2. Most of the patients with *in situ* disease had HER expression, with 16/21 having HER2 overexpression, 2/21 luminal HER2 and 3/21 triple-negative.

Surgery was performed in all the patients: mastectomy in 72.9% of cases and breast-conserving surgery (quadrantectomy) in 27.1%. Initially, oncoplastic breast surgery techniques were used in 69.4% of surgeries, with skin-sparing mastectomy being the most commonly used technique (36.5%), followed by the Grisotti plug-flap technique (24.7%). In the patients submitted to breast reconstruction, this was performed immediately in 64.7% of cases and later in 35.3%. Overall, 91.8% of the patients underwent some form of axillary surgery, with 55.3% being submitted to sentinel lymph node biopsy and 41.2% to axillary lymphadenectomy. In the six cases not submitted to axillary surgery, the disease was *in situ*. Most patients received adjuvant therapy, with radiotherapy being performed in 60% of cases. All the patients with estrogen receptor-positive disease were submitted to adjuvant hormone therapy. In those with invasive disease, chemotherapy was given in 75.0% of cases, while 69.8% of those with invasive disease and HER2 expression received trastuzumab. **Supplementary Table 4** show the results.

Differences in the characteristics of patients with a clinical diagnosis of PDB (erosion/ulceration/scaling of the nipple) were analyzed and compared to those with pathological/subclinical PDB, with PDB being confirmed at pathology in all cases. For this analysis, two separate groups were established (**Table 1**; **Supplementary Tables 1**, **4**). Clinical PDB was found to be associated with earlier TNM stages (p=0.028), and was normally associated with *in situ* carcinoma (p=0.033; p=0.045), and with patients undergoing breast-conserving surgery (p<0.001); however, these differences were not reflected in any difference in survival (p=0.275).

Mean follow-up time was 71.2 ± 43.3 months (range 13.7 to 174.6 months). Local recurrence rate was 4.7% (4 patients), with one of these patients having undergone breast-conserving surgery (the Grisotti plug-flap technique) and the others mastectomy, without reconstruction in one case, with skin-sparing reconstruction in another case and latissimus dorsi flap breast reconstruction in the remaining case. There were no cases of loss to follow-up; however, 15 patients died, in 9 cases from the cancer and in 6 cases from another cause. Overall actuarial survival at 60 and 120 months was 89.1% and 69.6%, respectively. Cancer-specific actuarial survival at 60 and 120 months was 92.3% and 83.1%, respectively (**Supplementary Figure 2**).

Evaluation of the factors associated with cancer-specific survival (**Table 2**; **Supplementary Table 5**; **Supplementary Figures 2**, **3**) showed TNM clinical stage to be the only variable associated with survival (p<0.001). In this sample, survival was not affected by the type of breast surgery (mastectomy or breast-conserving surgery), oncoplastic surgery or anti-HER2 therapy.

**Table 2:** Cancer-specified survival as a result of clinical variables.

Variable	Category	Survival		p
		60 months	120 months	
CSS	–	92,3	83,1	–
OS	–	89,1	69,6	–
Age	<40 years	77,1	77,1	0,917
	40 - 50 years	91,4	84,3	
	50 - 60 years	91,7	76,4	
	60 - 69 years	100	88,9	
Treatment	2006 - 2009	90,9	81,8	0,687
period	2010 - 2013	96,3	88,1	
	2014 - 2017	87,5	n/a	
	2018 - 2021	n/a	n/a	
Clinical PDB	Yes	98,0	86,9	0,275
	No	84,5	78,5	
Clinical Stage	0	100	92,9	< 0,001
TNM	I	100	100	
	II	86,3	77,6	
	III	85,7	64,3	
	IV*	0	0	
Prognostic	0	100	92,9	< 0,001
Stage- TNM	I	95,7	95,7	
	II	91,7	82,5	
	III	83,3	53,6	
	IV*	0	0	
Histological	Paget	83,3	83,3	0,190
subtype	Paget + DCIS	100	100	
	Paget + IC	86,6	74,6	
	Paget + DCIS + IC	100	000	
Molecular	DCIS	100	92,9	0,061
subtype	Lum. HER neg	62,5	41,7	
	Lum. B HER +	94,1	78,4	
	HER 2	90,5	90,5	
	Triple negative	100	80,0	

OS, overall survival; CSS, cancer-specific survival; DCIS, ductal carcinoma in situ; ID= invasive carcinoma.

n/a= not assessable (there are no patients with this follow-up time);

*death at 18 months follow-up.

## Discussion

The characteristics of the patients in the present series differ from those reported in other publications on PDB, particularly because 87.1% of the patients were diagnosed and treated after 2010, reflecting the major changes that have occurred in breast cancer management, including sentinel lymph node biopsy, oncoplastic surgery and the use of HER2-targeted therapies. Nevertheless, the high rate (92.9%) of association with DCIS and/or invasive ductal carcinoma reflects previous findings (13) and corroborates the fact that 57% of patients had a palpable lump at diagnosis.

A considerable number of studies have reported normal mammography findings in 22% to 50% of patients (15), thus concluding that the value of mammograms in defining the extent of the disease was limited. Conversely, in the present study, mammography detected some type of abnormality in over 87% of cases, showing it to be an important tool for evaluating the extent of underlying disease, considering the high number of patients with nodular lesions and micro-calcifications. The accuracy of diagnosis and of determining the extent of the disease can be improved by performing both breast ultrasonography and MRI. Here, ultrasonography detected a nodule in 72.2% of cases compared to 37% with mammography, highlighting the greater accuracy of that imaging test in detecting nodular lesions. Due to the multifocality of PDB, surgery has to be meticulously planned, requiring a combination of clinical examination, mammography and ultrasonography. MRI plays a role in the presurgical evaluation of patients who are candidates for breast-conserving surgery, since this imaging tool enables visualization of any underlying disease and its extent (15), particularly when mammography and ultrasonography results are negative (16). Nevertheless, the impact of MRI on treatment planning in cases of PDB has yet to be defined, since MRI would be indicated principally in the case of candidates for breast-conserving treatment (17) possibly increasing the indication for mastectomy. In this study, 23 patients were submitted to MRI of the breast, with 19 of them being initially considered candidates for mastectomy. MRI provided reassurance to convert to breast-conserving surgery in 6 cases (6/19, 31.6%), with 4 of these patients having tumors of 5 to 8 cm in size and the other two having multifocal disease. MRI of the breast may overestimate lesions, leading to unnecessary mastectomies, or, conversely, may underestimate lesions, resulting in breast-conserving surgery being performed instead (18); therefore, further studies on MRI in PDB are required.

Various studies on PDB have reported high rates of HER-2 positivity when underlying disease is present (3, 14), a finding that is in agreement with the results of the present study (73.5% of cases). HER-2 overexpression is associated with greater aggressiveness and poorer prognosis. However, the advances in anti-HER therapy have been well reported in the literature (16, 19). Since the present study consists of a historic series, not all the patients underwent anti-HER therapy and, although survival was better in the patients submitted to adjuvant treatment with trastuzumab, this difference was not statistically significant (p=0.500). Nevertheless, that finding could have been the result of the small sample size.

The surgical treatment of PDB depends on the site and extent of the disease. Breast-conserving treatment with resection of the central quadrant constitutes the principal treatment modality when the disease is limited to the areola. As a function of the extent of the disease and the presence of multifocality/multicentricity, mastectomy could be the treatment of choice (17), associated or not with breast reconstruction. According to the literature, the rate of breast-conserving treatment ranges from 3% to 38% of cases (9, 13, 17, 20). Various techniques, including the Grisotti technique, are used. Breast-conserving surgery, together with radiotherapy for patients with invasive or *in situ* disease, has become the preferred treatment (6). Depending on the extent and multifocality/multicentricity, mastectomy may be necessary (17), associated or not with immediate reconstruction performed with the use of a prosthesis or with locoregional flaps such as the latissimus dorsi myocutaneous flap (13, 21). The most commonly used technique in this study was skin-sparing mastectomy with reconstruction using a prosthesis (36.5%). Immediate breast reconstruction was performed in 64.7% of cases.

The type of surgery does not affect survival, as previously reported (20) and confirmed with the patients in the present study (p=0.251). In this respect, oncoplastic breast surgery improves cosmetic outcome. In this study oncoplastic surgery had no effect on survival (p=0.785).

The indication of radiotherapy in cases of PDB is linked to its indications in breast cancer in view of the frequent multifocality. It is mandatory when breast-conserving treatment is performed (22), but this requirement may differ when the patient undergoes mastectomy. In this study, 60% of patients were submitted to radiotherapy; however, with no impact on survival.

The source of data for this study was the pathology database, which could have led to a certain bias in patient selection. In this respect, we took the precaution of separating the patients according to clinical or pathological findings as well as based on clinical condition (1). To the best of our knowledge, this methodology has not previously been used in other studies. However, this separation is of the utmost importance, since it affects multifocality (p=0.045) and clinical stage (p<0.001), and may also affect survival, particularly when evaluating population-based studies with large sample sizes. Patients with the disease confined to the areola have a better prognosis, with negative prognostic factors including the presence of palpable disease and metastatic disease (23). Today, the belief that PDB appears to determine poorer prognosis is being questioned (2, 3); however, multiple factors can influence results, including the way in which patients are selected, the association with invasive disease and clinical stage, as well as the presence of HER2-positivity. To minimize differences, a study was performed using paired variables; nevertheless, PDB remained associated with a poorer prognosis (3). Therefore, population-based studies should be viewed with caution, and paired case-control studies are required, particularly including use of anti-HER2 therapy.

Studies conducted with data from the Surveillance, Epidemiology, and End Results (SEER) program evaluated variables associated with prognosis in PDB. Regarding disease-specific survival, the prognostic factors were age, histological grade, association with *in situ* disease and invasive disease, and clinical stage (4). Two studies compare patients with PDB-invasive and PDB-*in-situ* with non-PDB. The selection was based on histopathology code. PDB disease have different characteristic’s than non-PDB. PDB invasive has a worse breast cancer specific survival when comparing to similar patients without PDB (2, 3). But the groups were not matched. In the present study, univariate analysis showed the principal prognostic factor to be clinical stage at presentation, a factor that is influenced by multifocality. The limited number of patients also restricts the ability of the study to identify other potential variables. PDB consists of various breast pathologies, requiring different treatments and rendering a more detailed analysis difficult. This sample, although expressive for this disease, does not allow further conclusions to be drawn. It is important to perform a matched case-control study, comparing patients with similar clinical stage, and comparing patients with same molecular subtype. It will be our next study.

There are certain limitations associated with this study, particularly its retrospective nature. Notwithstanding, this series is one of the largest on Brazilian patients to be published and the largest to include tumor subtypes, with a follow-up time that exceeds five years and surgical treatment that includes oncoplastic surgery. Another limitation is that immunohistochemistry was only performed on the principal tumor and not on all the tumors; however, only two *in situ* carcinomas were not evaluated using immunohistochemistry. Differences were found in molecular subtype between DCIS and invasive ductal carcinoma, as well as a high rate of multicentricity/multifocality and a strong association with HER2-positivity; therefore, further studies on the subject are required.

Regarding future perspectives, studies are required that separate patients with the clinical presentation of the disease from those with the subclinical/pathological form, as well as studies to evaluate the actual role of MRI of the breast, of oncoplastic treatment and the impact of the addition of targeted therapy in this group of patients. Furthermore, the ideal design should be paired matched case-control studies comparing patients of the same clinical stage and/or molecular subtype with and without PDB in order to evaluate the true effect of PDB on survival.

## Data availability statement

The raw data supporting the conclusions of this article will be made available by the authors, after institutional evaluation.

## Ethics statement

The studies involving humans were approved by Barretos Cancer Hospital Ethics Research Committee. The studies were conducted in accordance with the local legislation and institutional requirements. Written informed consent for participation was not required from the participants or the participants’ legal guardians/next of kin because it is not necessary based on a Brazilian National regulatory Ethics Committee.

## Author contributions

RP: Data curation, Formal Analysis, Investigation, Validation, Visualization, Writing – original draft, Writing – review & editing. IO: Data curation, Investigation, Resources, Visualization, Writing – review & editing. RV: Conceptualization, Formal Analysis, Funding acquisition, Methodology, Project administration, Supervision, Validation, Visualization, Writing – original draft, Writing – review & editing.
