# Supplementary material for: Are there clinical and subclinical/pathological forms of Paget’s disease of the breast?
Source: Front Oncol. 2023 Nov 28;13:1287882. doi: 10.3389/fonc.2023.1287882 (PMC10715306; doi:10.3389/fonc.2023.1287882)
Supplement: Supplementary file 1 [file DataSheet_1.pdf]

**Are there clinical and subclinical/pathological forms of Paget's disease of the breast?**

Supplementary material


| | | |
|---|---|---|
| S.Table 1 | Clinical-radiological variables……………………………………. | 2 |
| S.Table 2 | Relation between pathologic and prognostic TNM……………… | 4 |
| S.Table 3 | Comparison between invasive disease and molecular subtype ….... | 5 |
| S.Table 4 | Treatment carried out and evolution related to PDB ……………… | 6 |
| S.Table 5 | Cancer-specific survival as a function of treatment type ………….. | 7 |
| S.Figure 1 | Clinical presentations of PDB …………………………………….. | 8 |
| S.Figure 2 | Survival: (a) Overall; (b) cancer specific survival ………….......... | 9 |
| S.Figure 3 | Cancer-specific survival (a) Clinical PDB; (b) pathological evaluation; (c) clinical stage TNM (d) prognostic stage TNM; (e) molecular subtype; (f) anti-HER therapy………………………….. | 10 |

**Supplementary Table** 1. Clinical-radiological variables

| Variable | Category | PDB clinical | | | p |
|---|---|---|---|---|---|
| | | Present | Absent | Total | |
| **Clinical presentation** | | | | | |
| Eczema* | Absent | 15 | 35 | 50 (58.8) | <0.001 |
| | Present | 35 | 0 | 35 (41.2) | |
| Bleeding | Absent | 40 | 32 | 72 (84.7) | 0.222 |
| | Present | 10 | 3 | 13 (15.3) | |
| Tumor | Absent | 36 | 8 | 44 (51.8) | <0.001 |
| | Present | 14 | 27 | 41 (48.2) | |
| **Physical exam** | | | | | |
| Eczema* | Absent | 20 | 35 | 55 (64.7) | < 0.001 |
| | Present | 30 | 0 | 30 (35.3) | |
| Ulceration* | Absent | 26 | 35 | 61 (71.8) | < 0.001 |
| | Present | 24 | 0 | 24 (28.2) | |
| Peeling* | Absent | 26 | 35 | 61 (7.8) | < 0.001 |
| | Present | 24 | 0 | 24 (28.2) | |
| Tumor | Absent | 30 | 7 | 37 (43.5) | < 0.001 |
| | Present | 20 | 28 | 48 (56.5) | |
| **Mammography** | | | | | |
| Normal | Absent | 37 | 27 | 64 (87.7) | 0.303 |
| | Present | 7 | 2 | 9(12.3) | |
| Skin thickening | Absent | 34 | 28 | 62 (84.9) | 0.041 |
| | Present | 10 | 1 | 11 (15.1) | |
| Malignant calcification | Absent | 21 | 9 | 30 (41.1) | 0.224 |
| | Present | 23 | 20 | 43 (58.9) | |
| Nodulation | Absent | 29 | 17 | 46 (63.0) | 0.622 |

| | | | | | |
|---|---|---|---|---|---|
| | Present | 15 | 12 | 27 (37.0) | |
| Asymmetry | Absent | 35 | 23 | 58 (79.5) | 1.000 |
| | Present | 9 | 6 | 15 (20.5) | |
| Architectural distortion | Absent | 44 | 27 | 71 (97.3) | 0.154 |
| | Present | 0 | 2 | 2 (2.7) | |
| **Ultrasonography** | | | | | |
| Microcalcification | Absent | 43 | 29 | 72 (91.1) | 0.133 |
| | Present | 2 | 5 | 7 (8.9) | |
| Nodulation | Absent | 16 | 6 | 22 (27.8) | 0.127 |
| | Present | 29 | 28 | 57 (72.2) | |
| NAC thickening | Absent | 37 | 33 | 70 (88.6) | 0.007 |
| | Present | 8 | 1 | 9 (11.4) | |

*Criteria used in the definition of clinical PDB; NAC - Nipple Areolar Complex

**Supplementary Table 2.** Relation between pathologic and prognostic TNM (p<0,001)

| Prog<br>Clinic | 0 | I | II | III | IV | All |
|---|---|---|---|---|---|---|
| 0 | 23 | 0 | 0 | 0 | 0 | 23 (27,1%) |
| I | 0 | 19 | 0 | 1 | 0 | 20 (23,5%) |
| II | 0 | 4 | 13 | 0 | 0 | 17 (20,0%) |
| III | 0 | 1 | 4 | 19 | 0 | 24 (28,2%) |
| IV | 0 | 0 | 0 | 0 | 1 | 1 (1,2%) |
| All | 23 (27,1%) | 24 (28,2%) | 17 (20,0%) | 20 (23,5%) | 1 ( 1,2%) | 85 (100,0%) |

Prog= prognostic TNM; Clinic= clinical TNM

**Supplementary Table 3.** Comparison between invasive disease and molecular subtype. (p=0.018)

| | CDIS | Invasive carcinoma | Total |
|---|---|---|---|
| Luminal B Her - | 0 | 12 (19.4%) | 12 (14.5%) |
| Luminal B Her + | 2 (9.5%) | 17 (27.4%) | 19 (22.9%) |
| Her2 + | 16 (76.2%) | 26 (41.9%) | 42 (50.6%) |
| Triple negative | 3 (14.3%) | 7 (11.3%) | 10 (12.0%) |
| Total | 21 (25.3%) | 62 (74.7%) | 83 (100.0%) |

* Two cases without information excluded

**Supplementary Table 4.** Treatment carried out and evolution related to PDB

| Variable | Category | Clinical PDB | | | p |
|---|---|---|---|---|---|
| | | Present | Absent | Total | |
| **Treatment** | | | | | |
| Breast Surgery | Mastectomy | 30 | 32 | 62 (72,9%) | < 0,001 |
| | BCS | 20 | 3 | 23 (27,1%) | |
| Initial OS | Present | 33 | 22 | 55 (64,7) | 0,821 |
| | Absent | 17 | 13 | 30 (35,3%) | |
| Axillary Surgery | AL | 17 | 15 | 32 (37,6%) | 0,97 |
| | SNB | 27 | 20 | 47 (55,3%) | |
| | Absent | 6 | 0 | 6 (7,1%) | |
| Chemotherapy* | Absent | 7 | 9 | 16 (25,0%) | 0,774 |
| | Present | 25 | 23 | 48 (75,0) | |
| Radiotherapy** | Absent | 17 | 17 | 34 (40,0%) | 0,187 |
| | Present | 33 | 18 | 51 (60,0%) | |
| Her2 Therapy# | Absent | 6 | 7 | 13 (30,2%) | 0,509 |
| | Present | 18 | 12 | 30 (69,8%) | |
| **Follow-up** | | | | | |
| Local recurrence | Absent | 47 | 34 | 81 (95,3%) | 0,640 |
| | Present | 3 | 1 | 4 (4,7%) | |
| Final status | LC | 41 | 28 | 69 (81,2%) | 0,296 |
| | LR | 0 | 1 | 1 (1,2%) | |
| | CRD | 4 | 5 | 9 (10,6%) | |
| | DAD | 5 | 1 | 6 (7,1%) | |

PDB= Paget Disease of the Breast; BCS= Breast Conservative Surgery; SNB= Sentinel Node of the Breast; AL: axillary lymphadenectomy; OS= Oncoplastic Surgery; LWC= Live without cancer; LWR= life with cancer recurrence; CRD= Cancer related death; DAD= Death associated to other disease

**Supplementary Table 5**. Cancer-specific survival as a function of treatment type

| Variable | Category | CSS | | p |
|---|---|---|---|---|
| | | 60 months | 120 months | |
| Breast surgery | Mastectomy | 89,6 | 79,9 | 0,251 |
| | BCS | 100 | 91,7 | |
| Initial OS | Present | 91,3 | 84,0 | 0,785 |
| | Absent | 93,3 | 81,6 | |
| Axillary surgery | AL | 86,1 | 74,8 | 0,227 |
| | SNB | 96,3 | 91,2 | |
| | Absent | 100 | 80,0 | |
| Chemotherapy* | Absent | 90,9 | 90,9 | 0,355 |
| | Present | 89,3 | 75,0 | |
| Radiotherapy** | Absent | 92,6 | 92,6 | 0,243 |
| | Present | 92,2 | 76,9 | |
| Her2 Therapy[#] | Her+ with TZM | 91,3 | 91,3 | 0,500 |
| | Her+ without TZM | 91,7 | 83,3 | |

CSS - Cancer-Specific Survival; BCS= Breast Conservative Surgery; SNB= Sentinel Node of the Breast; AL: axillary lymphadenectomy; OS= Oncoplastic Surgery; TZM= Trastuzumab; *Patients with invasive disease; # Patients with invasive disease Her 2 +

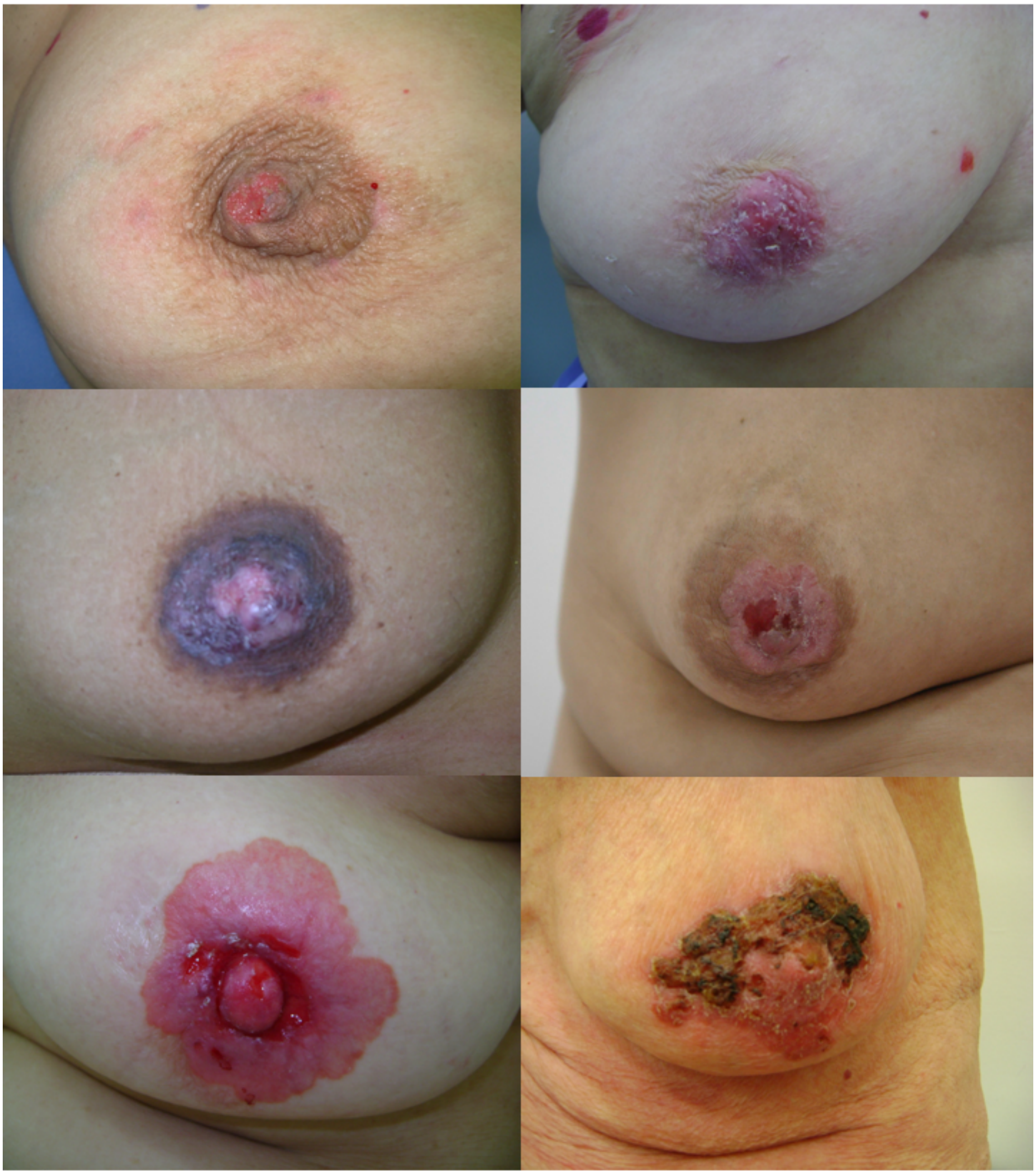

**Supplementary Figure 1.** Clinical presentations of Paget's disease of the breast

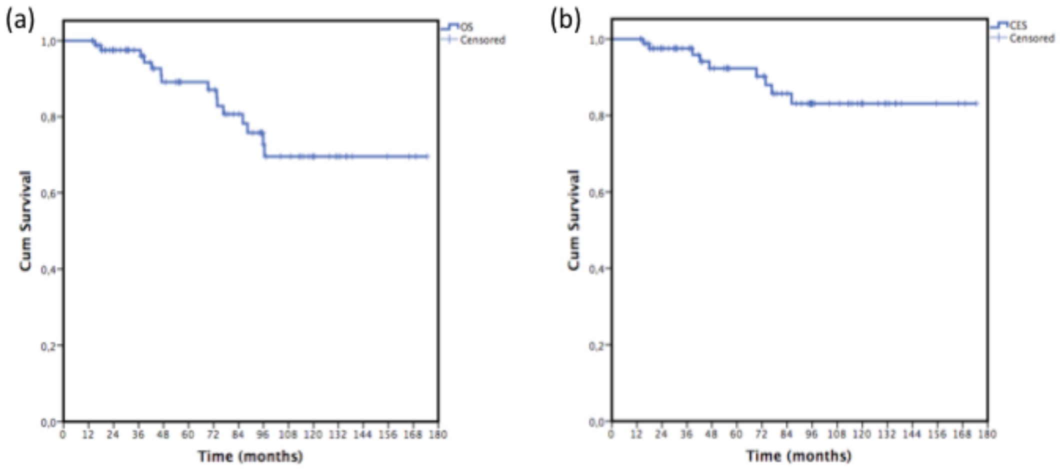


**Supplementary Figure 2**. Survival: (a) Overall; (b) cancer-specific survival

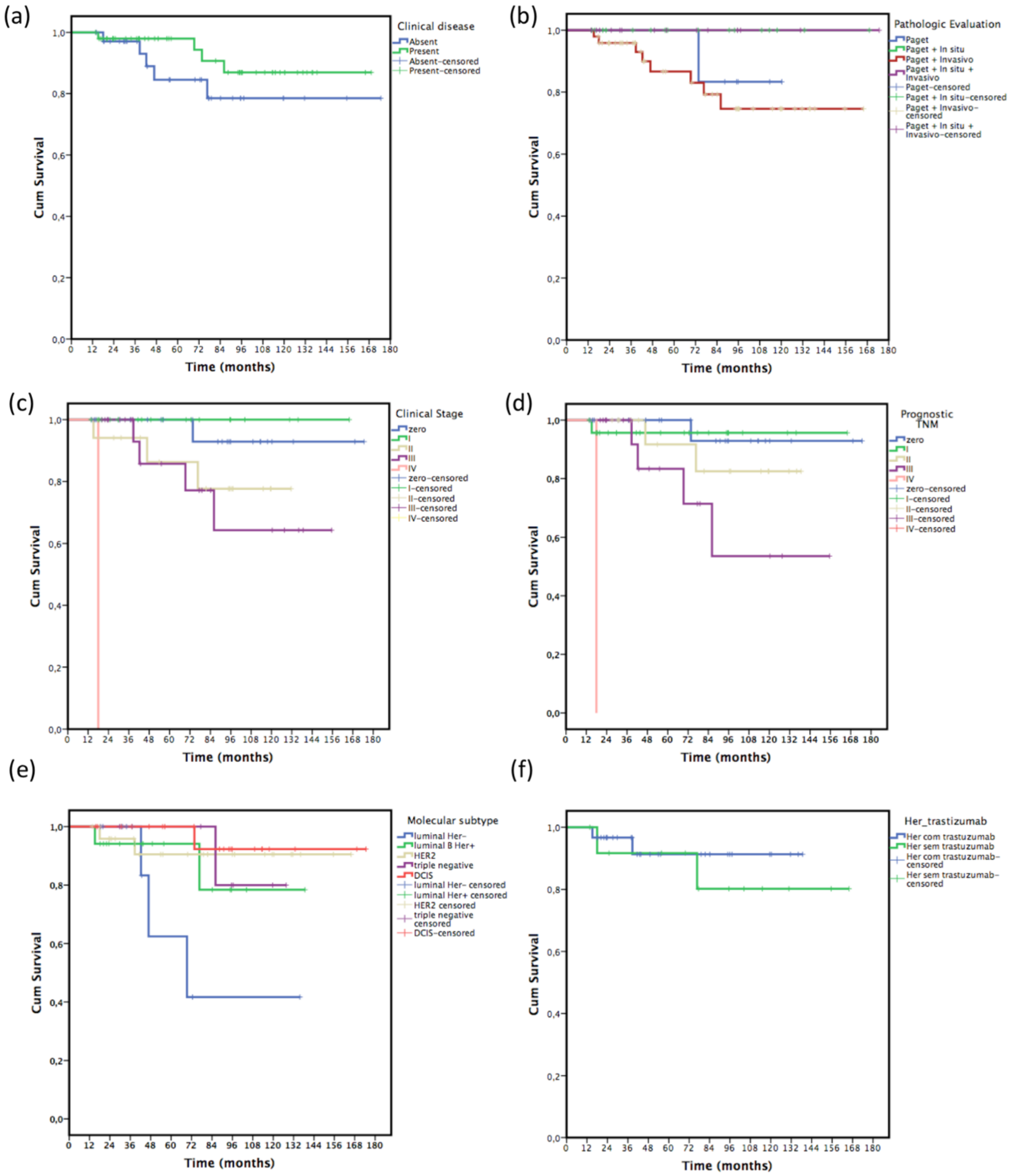


**Supplementary Figure 3.** Cancer-specific survival (a) Clinical PDB; (b) pathological evaluation; (c) clinical stage TNM (d) prognostic stage TNM; (e) molecular subtype; (f) anti-HER therapy
